# Clinical Significance of Lymphoscintigraphy Findings in the Evaluation of Lower Extremity Lymphedema

**DOI:** 10.4274/mirt.58077

**Published:** 2015-06-17

**Authors:** Seyhan Karaçavuş, Yunus Keser Yılmaz, Hasan Ekim

**Affiliations:** 1 Bozok University Faculty of Medicine, Department of Nuclear Medicine, Yozgat, Turkey; 2 Bozok University Faculty of Medicine, Department of Cardiovascular Surgery, Yozgat, Turkey

**Keywords:** lymphoscintigraphy, lymphedema, lower extremity

## Abstract

**Objective:**

The purpose of this study was to investigate the clinical significance of lymphoscintigraphy imaging in the evaluation of lower extremity lymphedema.

**Methods:**

Technetium-99m-labeled nanocolloid was injected subcutaneously in the first web spaces of both feet of 123 patients (M/F: 43/80, mean age 57.5±13.1 years, range 16-78 years) who had clinical evidence of lower extremity swelling with suspicion of lymphedema, and were referred for routine lymphoscintigraphy. Lymphoscintigraphy scan was started as dynamic viewing followed by static whole body imaging at 10 minute, 1 hour and 4 hours after injection.

**Results:**

Eighty-seven patients had lymphedema. Patients who had lymphedema were divided into two groups according to their scintigraphy findings: Group I included 58 patients without uptake in the popliteal nodes, and group II included 29 patients with positive popliteal nodes. The rate of popliteal node visualization was higher in patients with dermal backflow as compared to those without dermal backflow (p<0.001). The duration of lymphedema was also longer in patients with dermal backflow and popliteal nodes (p<0.004).

**Conclusion:**

Lymphoscintigraphy is a reliable, easily applied and well-tolerated objective method to diagnose lower extremity lymphedema. Uptake by popliteal lymph nodes and the presence of dermal backflow on lymphoscintigraphy, which is performed for evaluation of the lower limb lymphedema, were important signs indicating longer disease duration and higher severity of lymphatic dysfunction.

## Abstract (tr)

**Amaç:**

Bu çalışmada alt ekstremite lenfödeminin değerlendirilmesinde lenfosintigrafik bulguların öneminin araştırılması amaçlandı.

**Yöntem:**

Klinik olarak alt ekstremite şişliği ve lenfödem şüphesi mevcut olan, lenfosintigrafi için yönlendirilen 123 hastanın (E/K: 43/80, yaş ortalaması 57,5±13,1, yaş aralığı 16-78) her iki ayak birinci ve ikinci parmaklar arasından ciltaltına Tc-99m-nanokolloid enjekte edildi. Enjeksiyonun hemen ardından dinamik lenfosintigrafik görüntüleme başlatıldı, daha sonra 10. dk, 1. ve 4. saatlerde statik tüm vücut görüntülemeler yapıldı.

**Bulgular:**

Seksenyedi hastada lenfödem mevcuttu. Lenfosintigrafik tarama bulgularına gore lenfödemli hastalar iki gruba ayrıldı: Grup 1 popliteal lenf nodu gözlenmeyen 58 hastayı, grup 2 popliteal lenf nodu tutulumu gözlenen 29 hastayı içermekteydi. Popliteal lenf nodları dermal geri akım olan hastalarda olmayanlara gore daha fazla gözlendi (p<0,001). Lenfödem süresi, dermal geri akım ve popliteal lenf nodu gözlenen hastalarda daha uzundu (p<0,004).

**Sonuç:**

Alt ekstremite lenfödeminin tanısında lenfosintigrafi güvenilir, kolay uygulanan ve iyi tolere edilen objektif bir görüntüleme tekniğidir. Lenfosintigrafide popliteal lenf nodu tutulumu ve dermal geri akım varlığı, lenfatik disfonksiyonun ciddiyetinin ve daha uzun süreli hastalık mevcudiyetinin önemli işaretlerindendir.

## INTRODUCTION

The basis of lymphoscintigraphy is estimating the uptake of a radiolabelled tracer that is injected into the periphery and transported into the regional lymph nodes by the lymphatic system, and it is routinely performed as part of evaluation of a swollen limb (1). This technique might both determine the underlying cause of swelling and indicate its pathophysiology (2).

When lymphatic transport disrupts because of an injury to the lymphatics, infection or congenital abnormality, lymph-edema consists of excess tissue protein, edema, chronic inflammation and fibrosis within the skin and subcutaneous tissue resulting from anatomical or functional lymphatic obstruction (3,4). The diagnosis of lymphedema is usually made clinically through the presence of characteristic tissue swelling (5). However, particularly in its early stages, the differential diagnosis of this entity from other common causes of limb edema such as chronic venous insufficiency, deep vein thrombosis, cardiac failure, myxedema, and protein-losing conditions is crucial.

Lymphoscintigraphy is a credible, simple, reproducible, well tolerated and an objective method to diagnose and illustrate the severity of lymphedema. In this study, we aimed to investigate the clinical significance of lymphoscintigraphy findings in the evaluation of lower extremity lymphedema.

## MATERIALS AND METHODS

The study included 123 consecutive patients (M/F: 43/80, mean age 57.5±13.1 years, range 16-78 years) who were referred to Bozok University Hospital, Nuclear Medicine Department for evaluation of lower extremity swelling that was suspicious for lymphedema, from January 2011 to January 2015. The local Institutional Ethical Committee approved data collection and reviewing of images.

### Lymphoscintigraphy Study

**Figure 1:**
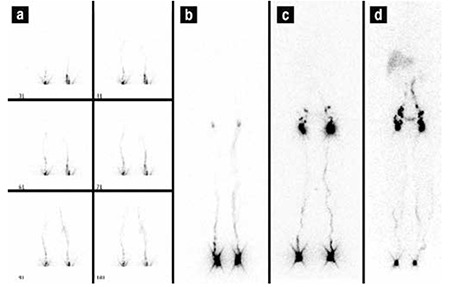
Lymphoscintigraphic findings of a normal subject. a) dynamic images b) 10th minute static whole body imaging c) 1st hour static imaging d) 1st hour static imaging

The patient was placed in supine position under a large field gamma camera (Philips Medical Systems Brightview Gamma Diagnost, Best, Holland), which included a low-energy general purpose collimator set at 140 KeV with 20% window, zoom 1.0 with 256×256 matrix size. The lymphoscintigraphy studies were performed by subcutaneous injection of 20 MBq (0.5 mCi) of technetium-99m-labeled nanocolloid (Senti-Scint; MEDI-Radiopharma, Budapest, Hungary) in a volume of 0.1 mL, using 26-gauge needle into the webbed spaces of both feet. The injection sites were massaged for 30 sec. following injection. Immediately after the injection, dynamic images were obtained for 2 min and static whole body imaging were recorded at 10th min, 1st, 4th and 24th hours when required (Figure 1). Patients were asked to take a short walk between the early and delayed scans without any strong exercise.

Lymphoscintigraphic interpretation included evaluation of the injection site (delay, presence or absence of lymphatic transport), lymphatic vessels (asymmetric visualization), collateral vessels, dermal backflow, lymph nodes (number, size and reduced, faint or no uptake of radiotracer) or presence of lymph nodes in the deep lymphatic system (i.e., popliteal node was considered positive when lymphoscintigraphy showed at least one discrete focus of activity at the level of the knee, between the injection site and the ipsilateral draining ilioinguinal lymph nodes) and abnormal radiotracer accumulation suggestive of extravasation, lymphocele, or lymphangiectasia. Lymphoscintigraphic staging was carried out for all patients as described by Lee and Bergan in Table 1 (6).

### Statistical Analysis

Continuous variables were expressed as mean ± standard deviation and categorical variables were expressed as frequency (percentage). Differences in continuous variables were evaluated using the Student’s t-test and the Chi-squared test was used to evaluate categorical variables. The correlation between parameters was analyzed using Spearman correlation tests. Correlation coefficient was depicted as r. All statistical calculations were performed using SPSS (Statistical Package for the Social Science; SPSS Inc., Chicago, IL, USA) version 18 for Microsoft Windows. The threshold of statistical significance was set at p<0.05.

## RESULTS

The mean age of 87 patients with lymphedema was 55.7±14.1; ranging between 16-78 years (34 men (39%), aged 57.7±14.7 years; 53 women (61%), aged 54.1±13.6 years). The mean duration of lower extremity swelling was 5.2±3.9 years.

Lymphoscintigraphy was performed in 246 extremities. Out of the 123 subjects with lower extremity swelling, 87 cases (71%) were found to be related to unilateral or bilateral lymphedema, due to either primary or secondary causes with clinical evidence of different levels of edema. The majority of patients (47, 54%) had primary lymphedema. Based on lymphoscintigraphy staging (L-stage), 30 cases (35%) had Stage I, 26 cases (30%) Stage II, 22 cases (25%) Stage III and the remaining 9 (10%) cases had Stage IV lymphedema. Table 2 shows the demographic and lymphoscintigraphic features of all patients.

**Figure 2:**
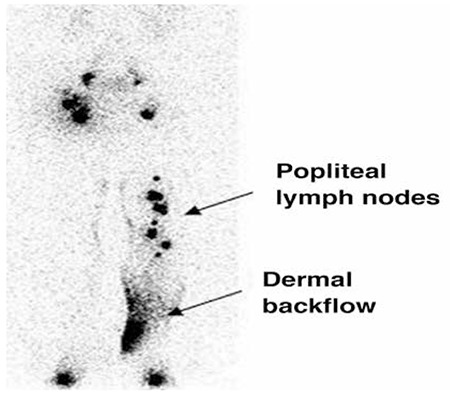
The popliteal lymph nodes and dermal back-flow as seen in a patient with left lower extremity lymph-edema for 8 years

The patients were divided into two groups; group 1 included 58 patients without popliteal node visualization on lymphoscintigraphy, while group 2 included 29 patients with positive popliteal node uptake (Table 3). Patients in group 2 had a significantly higher duration of lower extremity swelling (7.4±3.7 years) as compared to that of patients in group 1 (4.3±3.6 years) (p=0.003). Popliteal nodes were visualized in patients with dermal backflow more frequently than those without dermal backflow (p<0.0001). The duration of the disease was also longer in patients with dermal backflow (p<0.004). These findings showed a high positive correlation between the presence of dermal backflow or popliteal node uptake and severity of lymphatic dysfunction. Figure 2 demonstrates popliteal lymph nodes and dermal backflow in patients with left lower extremity lymphedema.

Popliteal nodes were observed in 21/48 patients (44%) with dermal backflow, however, they were not visualized in 31/39 patients (80%) without dermal back flow. There was significant correlation between dermal backflow and popliteal node visualization (r=0.6, p <0.001). The popliteal node uptake was higher in L-stage II and III as compared to L-stage I and IV (n=21, n=8, respectively; p<0.001).

## DISCUSSION

Lymphedema is a common, chronic and debilitating disease. Its diagnosis is challenging, especially in the early stages. Early diagnosis can provide alternatives for therapy plans and prevention of secondary problems that include lipid deposition, extremity deformity, and recurrent infections (3). Lymphoscintigraphy confirms lymphatic failure at any stage whenever the results are abnormal, as well as providing useful information about the pathophysiology and mechanism of failure (1). It evaluates lymphatic drainage pathways and distinguishes lymphatic pathology from non-lymphatic causes such as venous edema, myxedema, and lipedema. In addition, as Szuba et al. emphasized, patients with clinical suspicion of lymphedema were still referred to lymphoscintigraphy in order to confirm normal lymphatic flow (1). Sherman et al. first reported that radioactive colloidal gold (Au-198) had a potential for lymphatic system imaging in 1953 (7). Then, plasma proteins radiolabeled with I-131 was used for lymphatic system scanning, but it was not suitable for use due to high-energy irradiation emitters (8). The development of Tc-99m labeled radiocolloids and macromolecules has made lymphoscintigraphy a reliable, simple and practical technique (1).

Findings for lymphatic insufficiency include delay or absence of lymphatic transport from injection site, asymmetric or absent visualization of regional lymph nodes, and the presence of radiotracer uptake in dermal lymphatics called dermal backflow (9). Lymph nodes in the deep lymphatic system (i.e., popliteal lymph nodes), collateral lymphatic channels, dilated lymphatic vessels and interrupted vascular structures can be seen on lymphoscintigraphy (10). In addition, quantitative analysis improves the sensitivity and specificity of lymphoscintigraphy in diagnosing lymphedema (11,12,13). Lymphoscintigraphy has been also preferred in the evaluation of therapy for lymphedema (14,15).

**Figure 3:**
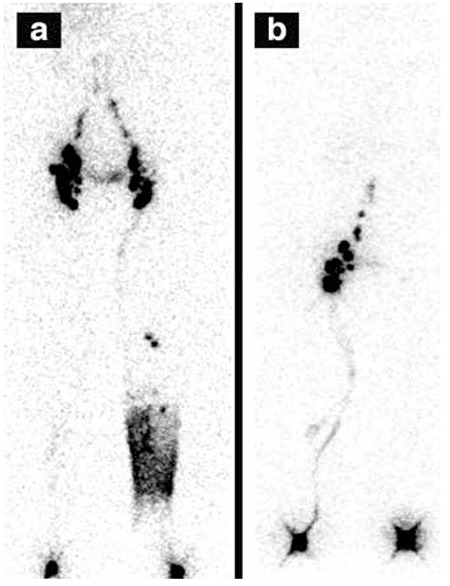
a) Lymphoscintigraphy findings of a patient at L-stage IIa; dermal back-flow and popliteal lymph nodes were seen on the left lower extremity. b) A patient at L-stage IV; without crossing the main lymphatic channels on left lower extremity. Without crossing the main lymphatic channels on left lower extremity

**Figure 4:**
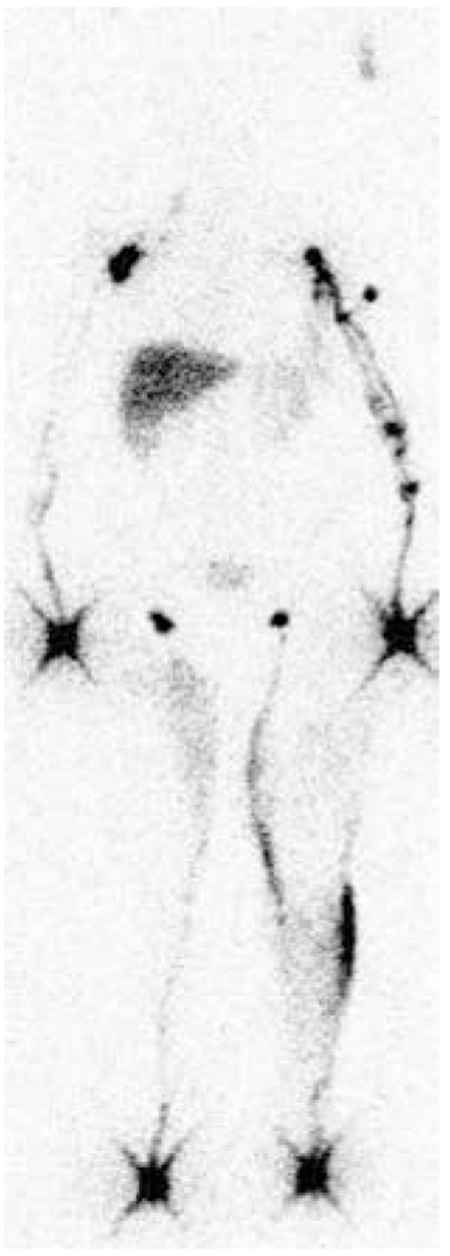
Lymphoscintigraphic findings of the patient with chylothorax of unclear etiology. Abnormal tracer accumulation was seen in the left thorax

**Figure 5:**
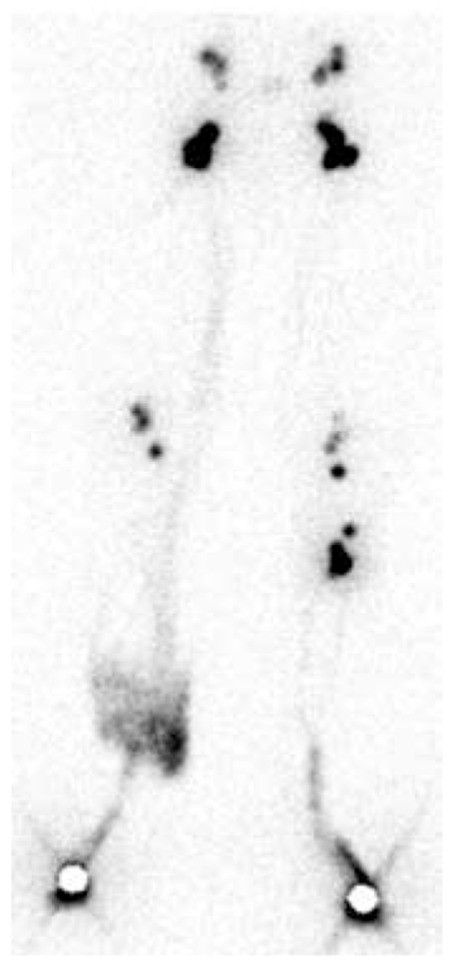
Lymphoscintigraphy of a 56-year-old woman diagnosed with bilateral lymphedema and documented venous disease and lymphangitis. Bilateral popliteal lymph nodes and focal tracer accumulation due to dermal backflow and lymphangitis were visible in the right lower extremity

In this study, we identified that the presence of dermal backflow and popliteal lymph nodes demonstrated lymphatic flow impairment, and they were associated with duration of lymphedema, which was longer in patients with dermal backflow and popliteal lymph nodes. Looking at the literature, Burnand et al pointed out in their study that popliteal nodes were observed in patients with abnormalities, and there was a strong relationship between dermal backflow and popliteal lymph node visualization (16). Similarly, Kandeel et al. reported that popliteal lymph node uptake during lymphoscintigraphy indicated lymph flow re-routing from the superficial to the deep system, and it was related to longer duration of lymphatic dysfunction (17). Our results were similar to previous studies. In L-stage II and III, these findings were seen more frequently as compared to L-stage I that represents early stage lymphedema and L-stage IV without crossing the main lymphatic channels and with absence of collateral lymphatic channels. As a matter of fact, lymphoscintigraphic staging is based on lymphoscintigraphic findings such as main lymphatics, dermal backflow and collaterals. Lee and Bergan developed new clinical and laboratory staging systems; the former was based on subjective and objective findings of local and systemic conditions, and the latter on lymphoscintigraphy findings aiming to improve the clinical management of chronic lymphedema (6). They suggested that these two staging systems are not just a new guideline to improve lymphedema management but also to provide a better prediction of treatment outcome, and to guide additional medical and surgical therapy planning. We also believe that standardized documentation of findings is essential for objective assessment, management and follow-up. Figure 3 demonstrates lymphoscintigraphy findings of patients at L-stage IIa and L-stage IV. When referring to a patient at this stage, the presence of a large amount of dermal backflow could be imagined in lymphoscintigraphy of this patient. In addition, abnormal tracer accumulation could be seen on lymphoscintigraphy in other diseases such as extravasation of lymphatic fluid into body cavities (chylothorax or chylous ascites), lymphocele, and lymphangiectasia (1). Figure 4 and 5 show our patients with chylothorax and lymphangitis, respectively.

## CONCLUSION

Lymphoscintigraphy is a reliable, easily applied, well-tolerated and accurate imaging technique in the diagnosis of lymphedema. Uptake by popliteal lymph nodes and the presence of dermal backflow on lymphoscintigraphy, which is performed for the evaluation of lower limb lymphedema, were important signs indicating longer disease duration and higher severity of lymphatic dysfunction. Lymphoscintigraphic staging could be used for objective assessment of lymphoscintigraphy studies.

## Figures and Tables

**Table 1:**
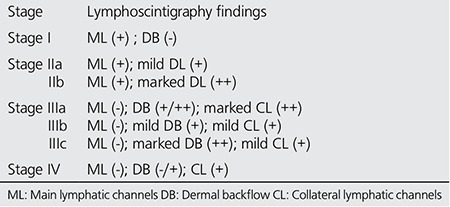
Lymphoscintigraphic staging (by Lee and Bergan)

**Table 2:**
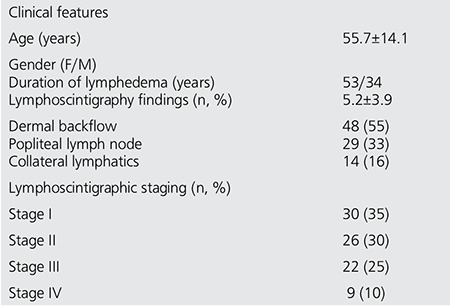
Demographic and lymphoscintigraphic characteristics of all patients (n=87)

**Table 3:**
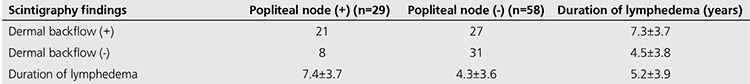
Visualization of dermal backflow and popliteal lymph nodes on the same extremity
